# 4-[(4-Chloro­phen­yl)(phen­yl)­meth­yl]­piperazin-1-ium picrate monohydrate

**DOI:** 10.1107/S1600536812031984

**Published:** 2012-07-18

**Authors:** Yanxi Song, C. S. Chidan Kumar, S. Chandraju, S. Naveen, Hongqi Li

**Affiliations:** a School of Environmental Science and Engineering, Donghua University, Shanghai 201620, People’s Repulic of China; b Department of Chemistry, G. Madegowda Institute of Technology, Bharathi Nagar 571 422, India; c Department of Sugar Technology, University of Mysore, Sir. M. V. PG Center, Tubinakere 571 402, India; d Department of Physics, School of Engineering and Technology, Jain University, Bangalore 562 112, India; e College of Chemistry, Chemical Engineering and Biotechnology, Donghua University, Shanghai 201620, People’s Repulic of China

## Abstract

The asymmetric unit of the title compound, C_17_H_20_ClN_2_^+^·C_6_H_2_N_3_O_7_^−^·H_2_O, contains a piperazin-1-ium cation, a picrate anion and one solvent water mol­ecule. The piperazene ring is protonated at one N atom and adopts a highly distorted chair conformation with the chloro­pheny(phen­yl)methyl substituent on the second N atom in an equatorial position. The crystal structure is stabilized by O—H⋯O, N—H⋯O and C—H⋯O hydrogen bonds.

## Related literature

For the biological activity of 1-benzyl­piperazine, see: Campbell *et al.* (1973 ▶). For related structures, see: Jasinski *et al.* (2011 ▶); Song *et al.* (2012 ▶). For bond-length data, see: Allen *et al.* (1987 ▶).
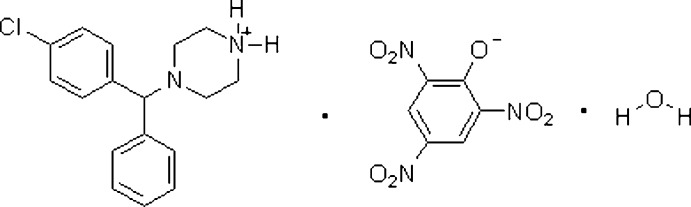


## Experimental

### 

#### Crystal data

C_17_H_20_ClN_2_^+^·C_6_H_2_N_3_O_7_^−^·H_2_O
*M* *_r_* = 533.92
Monoclinic, 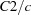

*a* = 21.144 (2) Å
*b* = 8.2997 (8) Å
*c* = 28.528 (3) Å
β = 93.029 (1)°
*V* = 4999.3 (8) Å^3^
*Z* = 8
Mo *K*α radiation
μ = 0.21 mm^−1^
*T* = 296 K
0.25 × 0.22 × 0.07 mm

#### Data collection

Bruker APEXII CCD diffractometer
Absorption correction: multi-scan (*SADABS*; Bruker, 2005 ▶) *T*_min_ = 0.949, *T*_max_ = 0.985
12567 measured reflections
4417 independent reflections
2996 reflections with *I* > 2σ(*I*)
*R*_int_ = 0.026

#### Refinement

*R*[*F* ^2^ > 2σ(*F* ^2^)] = 0.057
*wR*(*F* ^2^) = 0.175
*S* = 1.04
4417 reflections
334 parameters
H-atom parameters constrained
Δρ_max_ = 0.63 e Å^−3^
Δρ_min_ = −0.46 e Å^−3^

### 

Data collection: *APEX2* (Bruker, 2005 ▶); cell refinement: *SAINT* (Bruker, 2005 ▶); data reduction: *SAINT*; program(s) used to solve structure: *SHELXS97* (Sheldrick, 2008 ▶); program(s) used to refine structure: *SHELXL97* (Sheldrick, 2008 ▶); molecular graphics: *SHELXTL* (Sheldrick, 2008 ▶); software used to prepare material for publication: *SHELXTL*.

## Supplementary Material

Crystal structure: contains datablock(s) global, I. DOI: 10.1107/S1600536812031984/sj5246sup1.cif

Structure factors: contains datablock(s) I. DOI: 10.1107/S1600536812031984/sj5246Isup2.hkl

Supplementary material file. DOI: 10.1107/S1600536812031984/sj5246Isup3.cml

Additional supplementary materials:  crystallographic information; 3D view; checkCIF report

## Figures and Tables

**Table 1:** Hydrogen-bond geometry (Å, °)

*D*—H⋯*A*	*D*—H	H⋯*A*	*D*⋯*A*	*D*—H⋯*A*
N2—H2*A*⋯O1*W* ^i^	0.90	2.03	2.850 (4)	151
N2—H2*B*⋯O1^ii^	0.90	1.96	2.819 (3)	160
N2—H2*B*⋯O2^ii^	0.90	2.42	3.017 (4)	124
O1*W*—H1*WA*⋯O1	0.85	1.97	2.805 (3)	168
C16—H16*B*⋯O5^iii^	0.97	2.34	3.166 (4)	143

Symmetry codes: (i) 
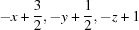
; (ii) 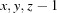; (iii) 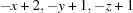.

**Fig. 1.**
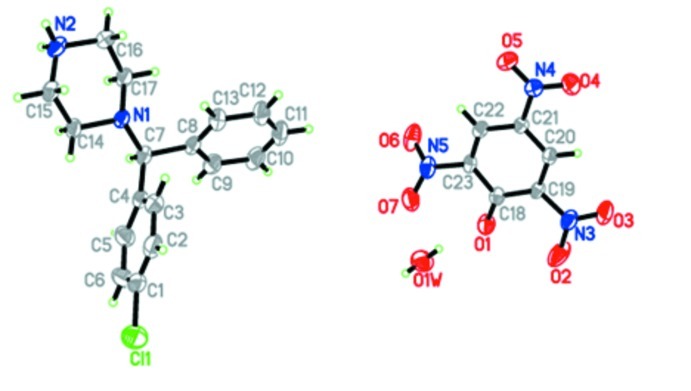
A view of the title compound with the atom numbering scheme. Displacement ellipsoids for non-H atoms are drawn at the 50% probability level.

## supplementary crystallographic information

### Comment

1-Benzylpiperazine was originally synthesized as a potential anthelmintic (Campbell *et al.*, 1973) and its derivatives were found to possess excellent pharmacological activities. These include vasodilator, hypotensive and antiviral activity, the ability to increase cerebral blood flow and broad pharmacological action on central nervous system. In the course of our studies on the salts of piperazines (Jasinski *et al.*, 2011; Song *et al.*, 2012) and in view of the importance of piperazines, the paper reports the crystal and molecular structure of the title piperazin-1-ium salt.

The molecular structure and atom numbering scheme of the title compound are shown in Fig 1. In the title compound, the piperazine group is protonated at the N2 atom and adopts a highly distorted chair conformation with puckering parameters Q, θ and φ having values of 0.595 (3) °, 6.4 (3) ° and 342 (3) °, respectively. For an ideal chair conformation, θ has a value of 0 or 180°. The bond lengths (Allen *et al.*, 1987) and bond angles are in good agreement with standard values. The crystal structure is stabilized by intermolecular O–H···O, N–H···O and C–H···O hydrogen bonds.

### Experimental

1-((4-Chlorophenyl)(phenyl)methyl)piperazine (2.88 g, 0.01 mol) and picric acid (2.99 g, 0.01 mol) were dissolved separately in methanol. Both the solutions were mixed together and stirred for a few minutes at room temperature. The precipitate was collected by filtration and purified by recrystallization from methanol. On recrystallization with DMF after 15 days, good quality single crystals were obtained by the slow evaporation method. (M.P.: 441–445 K).

### Refinement

H atoms were placed at idealized positions and allowed to ride on their parent atoms with C—H distances in the range 0.92–0.98 Å and N—H = 0.86 Å; *U*_iso_(H) values were set equal to 1.2U_eq_(carrier atom).

### Crystal data

**Table:**

C_17_H_20_ClN_2_^+^·C_6_H_2_N_3_O_7_^−^·H_2_O	*F*(000) = 2224
*M**_r_* = 533.92	*D*_x_ = 1.419 Mg m^−^^3^
Monoclinic, *C*2/*c*	Melting point = 441–445 K
Hall symbol: -C 2yc	Mo *K*α radiation, λ = 0.71073 Å
*a* = 21.144 (2) Å	Cell parameters from 2514 reflections
*b* = 8.2997 (8) Å	θ = 2.3–21.0°
*c* = 28.528 (3) Å	µ = 0.21 mm^−^^1^
β = 93.029 (1)°	*T* = 296 K
*V* = 4999.3 (8) Å^3^	Block, yellow
*Z* = 8	0.25 × 0.22 × 0.07 mm

### Data collection

**Table:**

Bruker APEXII CCD diffractometer	4417 independent reflections
Radiation source: fine-focus sealed tube	2996 reflections with *I* > 2σ(*I*)
Graphite monochromator	*R*_int_ = 0.026
φ and ω scans	θ_max_ = 25.1°, θ_min_ = 2.3°
Absorption correction: multi-scan (*SADABS*; Bruker, 2005)	*h* = −25→20
*T*_min_ = 0.949, *T*_max_ = 0.985	*k* = −9→8
12567 measured reflections	*l* = −31→33

### Refinement

**Table:**

Refinement on *F*^2^	Primary atom site location: structure-invariant direct methods
Least-squares matrix: full	Secondary atom site location: difference Fourier map
*R*[*F*^2^ > 2σ(*F*^2^)] = 0.057	Hydrogen site location: inferred from neighbouring sites
*wR*(*F*^2^) = 0.175	H-atom parameters constrained
*S* = 1.04	*w* = 1/[σ^2^(*F*_o_^2^) + (0.0837*P*)^2^ + 4.3115*P*] where *P* = (*F*_o_^2^ + 2*F*_c_^2^)/3
4417 reflections	(Δ/σ)_max_ < 0.001
334 parameters	Δρ_max_ = 0.63 e Å^−^^3^
0 restraints	Δρ_min_ = −0.46 e Å^−^^3^

### Special details

**Table:**

Geometry. All e.s.d.'s (except the e.s.d. in the dihedral angle between two l.s. planes) are estimated using the full covariance matrix. The cell e.s.d.'s are taken into account individually in the estimation of e.s.d.'s in distances, angles and torsion angles; correlations between e.s.d.'s in cell parameters are only used when they are defined by crystal symmetry. An approximate (isotropic) treatment of cell e.s.d.'s is used for estimating e.s.d.'s involving l.s. planes.
Refinement. Refinement of *F*^2^ against ALL reflections. The weighted *R*-factor *wR* and goodness of fit *S* are based on *F*^2^, conventional *R*-factors *R* are based on *F*, with *F* set to zero for negative *F*^2^. The threshold expression of *F*^2^ > σ(*F*^2^) is used only for calculating *R*-factors(gt) *etc*. and is not relevant to the choice of reflections for refinement. *R*-factors based on *F*^2^ are statistically about twice as large as those based on *F*, and *R*- factors based on ALL data will be even larger.

### Fractional atomic coordinates and isotropic or equivalent isotropic displacement parameters (Å^2^)

**Table:**

	*x*	*y*	*z*	*U*_iso_*/*U*_eq_	
C1	0.4904 (2)	0.8333 (6)	0.18443 (11)	0.0783 (11)	
C2	0.5527 (2)	0.8799 (4)	0.19014 (11)	0.0737 (10)	
H2	0.5633	0.9873	0.1955	0.088*	
C3	0.59944 (16)	0.7637 (4)	0.18770 (10)	0.0606 (8)	
H3	0.6418	0.7938	0.1915	0.073*	
C4	0.58414 (13)	0.6039 (4)	0.17965 (9)	0.0490 (7)	
C5	0.52105 (16)	0.5623 (5)	0.17391 (12)	0.0700 (9)	
H5	0.5100	0.4553	0.1682	0.084*	
C6	0.47386 (18)	0.6774 (7)	0.17651 (14)	0.0917 (13)	
H6	0.4314	0.6481	0.1729	0.110*	
C7	0.63529 (13)	0.4768 (3)	0.17948 (9)	0.0471 (7)	
H7	0.6158	0.3740	0.1699	0.057*	
C8	0.66452 (14)	0.4592 (3)	0.22959 (9)	0.0472 (7)	
C9	0.63449 (16)	0.3611 (4)	0.26056 (10)	0.0609 (8)	
H9	0.5991	0.3016	0.2503	0.073*	
C10	0.65702 (19)	0.3512 (4)	0.30690 (11)	0.0749 (10)	
H10	0.6356	0.2884	0.3279	0.090*	
C11	0.7106 (2)	0.4329 (4)	0.32234 (11)	0.0766 (11)	
H11	0.7259	0.4240	0.3534	0.092*	
C12	0.74098 (18)	0.5272 (4)	0.29149 (11)	0.0683 (9)	
H12	0.7776	0.5820	0.3015	0.082*	
C13	0.71784 (15)	0.5421 (3)	0.24554 (10)	0.0572 (8)	
H13	0.7385	0.6089	0.2251	0.069*	
C14	0.65583 (14)	0.5363 (4)	0.09816 (9)	0.0526 (7)	
H14A	0.6220	0.6154	0.0980	0.063*	
H14B	0.6379	0.4345	0.0874	0.063*	
C15	0.70566 (15)	0.5901 (4)	0.06551 (10)	0.0569 (8)	
H15A	0.6869	0.5989	0.0338	0.068*	
H15B	0.7213	0.6956	0.0750	0.068*	
C16	0.78429 (15)	0.4460 (4)	0.11470 (10)	0.0624 (8)	
H16A	0.8031	0.5443	0.1274	0.075*	
H16B	0.8170	0.3640	0.1148	0.075*	
C17	0.73229 (14)	0.3923 (4)	0.14476 (10)	0.0535 (7)	
H17A	0.7135	0.2937	0.1321	0.064*	
H17B	0.7495	0.3703	0.1763	0.064*	
C18	0.89491 (14)	0.6145 (3)	0.98892 (9)	0.0476 (7)	
C19	0.92823 (13)	0.7277 (3)	1.01969 (9)	0.0458 (6)	
C20	0.98723 (13)	0.7849 (3)	1.01257 (9)	0.0478 (7)	
H20	1.0065	0.8563	1.0340	0.057*	
C21	1.01856 (13)	0.7372 (3)	0.97362 (9)	0.0463 (6)	
C22	0.98998 (13)	0.6302 (3)	0.94120 (9)	0.0473 (7)	
H22	1.0111	0.5966	0.9151	0.057*	
C23	0.93097 (14)	0.5763 (3)	0.94855 (8)	0.0456 (6)	
Cl1	0.43227 (7)	0.9810 (2)	0.18712 (4)	0.1402 (6)	
N1	0.68382 (10)	0.5177 (2)	0.14618 (7)	0.0434 (5)	
N2	0.75908 (12)	0.4746 (3)	0.06606 (8)	0.0579 (7)	
H2A	0.7458	0.3808	0.0531	0.069*	
H2B	0.7900	0.5140	0.0488	0.069*	
N3	0.89901 (14)	0.7854 (3)	1.06134 (9)	0.0658 (7)	
N4	1.08105 (12)	0.7960 (3)	0.96648 (9)	0.0631 (7)	
N5	0.90198 (15)	0.4691 (3)	0.91305 (9)	0.0638 (7)	
O1	0.84301 (10)	0.5503 (3)	0.99573 (7)	0.0664 (6)	
O2	0.84210 (16)	0.7700 (4)	1.06424 (12)	0.1309 (14)	
O3	0.92957 (14)	0.8649 (4)	1.08951 (9)	0.1129 (11)	
O4	1.10570 (11)	0.8886 (3)	0.99550 (9)	0.0830 (7)	
O5	1.10730 (11)	0.7526 (4)	0.93145 (9)	0.0903 (8)	
O6	0.93478 (15)	0.3624 (3)	0.89779 (9)	0.0927 (9)	
O7	0.84749 (14)	0.4966 (4)	0.89915 (9)	0.0922 (9)	
O1W	0.74452 (12)	0.3604 (3)	0.95584 (11)	0.0996 (9)	
H1WA	0.7756	0.4210	0.9638	0.100*	
H1WB	0.7219	0.4009	0.9333	0.100*	

### Atomic displacement parameters (Å^2^)

**Table:**

	*U*^11^	*U*^22^	*U*^33^	*U*^12^	*U*^13^	*U*^23^
C1	0.076 (3)	0.113 (3)	0.0465 (18)	0.037 (2)	0.0071 (17)	0.008 (2)
C2	0.104 (3)	0.069 (2)	0.0476 (18)	0.020 (2)	0.0063 (18)	−0.0020 (16)
C3	0.063 (2)	0.066 (2)	0.0534 (17)	0.0055 (16)	0.0062 (14)	−0.0042 (15)
C4	0.0517 (17)	0.0633 (19)	0.0323 (13)	−0.0009 (14)	0.0060 (11)	0.0012 (12)
C5	0.057 (2)	0.086 (2)	0.066 (2)	−0.0055 (18)	−0.0042 (16)	0.0045 (18)
C6	0.053 (2)	0.139 (4)	0.083 (3)	0.013 (2)	−0.0016 (19)	0.021 (3)
C7	0.0550 (17)	0.0479 (16)	0.0390 (14)	−0.0083 (13)	0.0066 (12)	−0.0017 (12)
C8	0.0609 (18)	0.0451 (15)	0.0362 (14)	0.0050 (13)	0.0099 (12)	0.0016 (12)
C9	0.070 (2)	0.0621 (19)	0.0514 (17)	−0.0011 (16)	0.0138 (15)	0.0072 (15)
C10	0.102 (3)	0.078 (2)	0.0464 (18)	0.010 (2)	0.0210 (18)	0.0172 (17)
C11	0.114 (3)	0.074 (2)	0.0407 (17)	0.022 (2)	−0.0060 (18)	−0.0037 (17)
C12	0.092 (3)	0.059 (2)	0.0517 (19)	0.0035 (18)	−0.0103 (17)	−0.0110 (16)
C13	0.076 (2)	0.0493 (17)	0.0459 (16)	−0.0060 (15)	0.0015 (14)	0.0000 (13)
C14	0.0597 (18)	0.0622 (18)	0.0360 (14)	−0.0025 (14)	0.0050 (12)	0.0029 (13)
C15	0.068 (2)	0.0625 (19)	0.0413 (15)	−0.0056 (15)	0.0105 (13)	0.0058 (13)
C16	0.0562 (19)	0.077 (2)	0.0551 (18)	0.0061 (16)	0.0105 (14)	−0.0074 (16)
C17	0.0615 (18)	0.0558 (17)	0.0437 (15)	0.0042 (14)	0.0074 (13)	−0.0004 (13)
C18	0.0596 (18)	0.0456 (15)	0.0377 (14)	−0.0050 (13)	0.0036 (12)	0.0050 (12)
C19	0.0569 (18)	0.0463 (15)	0.0349 (13)	−0.0004 (13)	0.0105 (12)	−0.0017 (11)
C20	0.0556 (18)	0.0469 (15)	0.0407 (14)	0.0011 (13)	0.0000 (12)	−0.0056 (12)
C21	0.0450 (16)	0.0524 (16)	0.0416 (14)	0.0024 (12)	0.0037 (12)	−0.0032 (12)
C22	0.0587 (18)	0.0481 (16)	0.0357 (14)	0.0051 (13)	0.0066 (12)	0.0004 (12)
C23	0.0615 (18)	0.0420 (15)	0.0332 (13)	−0.0034 (13)	0.0002 (12)	−0.0011 (11)
Cl1	0.1339 (11)	0.1911 (15)	0.0945 (9)	0.1027 (11)	−0.0038 (7)	0.0031 (8)
N1	0.0497 (13)	0.0483 (13)	0.0329 (11)	0.0007 (10)	0.0079 (9)	0.0009 (9)
N2	0.0628 (16)	0.0653 (16)	0.0474 (14)	−0.0124 (13)	0.0204 (11)	−0.0090 (12)
N3	0.073 (2)	0.0739 (18)	0.0525 (15)	−0.0172 (15)	0.0207 (14)	−0.0167 (13)
N4	0.0517 (16)	0.0783 (18)	0.0599 (16)	−0.0023 (13)	0.0082 (13)	−0.0124 (14)
N5	0.083 (2)	0.0657 (18)	0.0430 (14)	−0.0191 (16)	0.0051 (14)	−0.0043 (13)
O1	0.0707 (15)	0.0780 (15)	0.0518 (12)	−0.0294 (12)	0.0165 (10)	−0.0060 (10)
O2	0.110 (2)	0.157 (3)	0.134 (3)	−0.068 (2)	0.080 (2)	−0.084 (2)
O3	0.096 (2)	0.176 (3)	0.0682 (16)	−0.019 (2)	0.0176 (14)	−0.0608 (19)
O4	0.0640 (15)	0.1024 (19)	0.0827 (16)	−0.0237 (14)	0.0053 (12)	−0.0275 (15)
O5	0.0620 (15)	0.131 (2)	0.0809 (17)	−0.0089 (15)	0.0277 (13)	−0.0341 (16)
O6	0.142 (3)	0.0651 (15)	0.0704 (16)	−0.0023 (16)	0.0001 (16)	−0.0268 (13)
O7	0.0768 (18)	0.135 (2)	0.0642 (16)	−0.0288 (17)	−0.0021 (13)	−0.0199 (15)
O1W	0.0735 (17)	0.0920 (19)	0.133 (2)	−0.0152 (14)	0.0024 (16)	−0.0241 (17)

### Geometric parameters (Å, º)

**Table:**

C1—C6	1.356 (6)	C15—H15B	0.9700
C1—C2	1.376 (5)	C16—N2	1.479 (4)
C1—Cl1	1.740 (4)	C16—C17	1.497 (4)
C2—C3	1.384 (5)	C16—H16A	0.9700
C2—H2	0.9300	C16—H16B	0.9700
C3—C4	1.382 (4)	C17—N1	1.463 (3)
C3—H3	0.9300	C17—H17A	0.9700
C4—C5	1.379 (4)	C17—H17B	0.9700
C4—C7	1.511 (4)	C18—O1	1.244 (3)
C5—C6	1.386 (5)	C18—C19	1.444 (4)
C5—H5	0.9300	C18—C23	1.449 (4)
C6—H6	0.9300	C19—C20	1.360 (4)
C7—N1	1.474 (3)	C19—N3	1.449 (4)
C7—C8	1.534 (4)	C20—C21	1.381 (4)
C7—H7	0.9800	C20—H20	0.9300
C8—C13	1.378 (4)	C21—C22	1.397 (4)
C8—C9	1.381 (4)	C21—N4	1.433 (4)
C9—C10	1.384 (4)	C22—C23	1.352 (4)
C9—H9	0.9300	C22—H22	0.9300
C10—C11	1.372 (5)	C23—N5	1.459 (4)
C10—H10	0.9300	N2—H2A	0.9000
C11—C12	1.364 (5)	N2—H2B	0.9000
C11—H11	0.9300	N3—O3	1.202 (3)
C12—C13	1.380 (4)	N3—O2	1.217 (4)
C12—H12	0.9300	N4—O5	1.223 (3)
C13—H13	0.9300	N4—O4	1.226 (3)
C14—N1	1.471 (3)	N5—O6	1.219 (4)
C14—C15	1.510 (4)	N5—O7	1.220 (4)
C14—H14A	0.9700	O1W—H1WA	0.8501
C14—H14B	0.9700	O1W—H1WA	0.8501
C15—N2	1.481 (4)	O1W—H1WB	0.8502
C15—H15A	0.9700		
			
C6—C1—C2	121.6 (3)	H15A—C15—H15B	108.0
C6—C1—Cl1	120.2 (4)	N2—C16—C17	110.2 (2)
C2—C1—Cl1	118.2 (4)	N2—C16—H16A	109.6
C1—C2—C3	118.7 (4)	C17—C16—H16A	109.6
C1—C2—H2	120.6	N2—C16—H16B	109.6
C3—C2—H2	120.6	C17—C16—H16B	109.6
C4—C3—C2	121.0 (3)	H16A—C16—H16B	108.1
C4—C3—H3	119.5	N1—C17—C16	109.9 (2)
C2—C3—H3	119.5	N1—C17—H17A	109.7
C5—C4—C3	118.5 (3)	C16—C17—H17A	109.7
C5—C4—C7	120.9 (3)	N1—C17—H17B	109.7
C3—C4—C7	120.6 (3)	C16—C17—H17B	109.7
C4—C5—C6	121.0 (4)	H17A—C17—H17B	108.2
C4—C5—H5	119.5	O1—C18—C19	126.1 (3)
C6—C5—H5	119.5	O1—C18—C23	122.4 (2)
C1—C6—C5	119.2 (4)	C19—C18—C23	111.4 (2)
C1—C6—H6	120.4	C20—C19—C18	123.9 (2)
C5—C6—H6	120.4	C20—C19—N3	116.2 (2)
N1—C7—C4	111.4 (2)	C18—C19—N3	119.9 (3)
N1—C7—C8	111.3 (2)	C19—C20—C21	120.2 (2)
C4—C7—C8	108.5 (2)	C19—C20—H20	119.9
N1—C7—H7	108.6	C21—C20—H20	119.9
C4—C7—H7	108.6	C20—C21—C22	120.4 (3)
C8—C7—H7	108.6	C20—C21—N4	120.1 (2)
C13—C8—C9	118.6 (3)	C22—C21—N4	119.5 (2)
C13—C8—C7	122.8 (2)	C23—C22—C21	118.6 (2)
C9—C8—C7	118.5 (3)	C23—C22—H22	120.7
C8—C9—C10	120.0 (3)	C21—C22—H22	120.7
C8—C9—H9	120.0	C22—C23—C18	125.4 (2)
C10—C9—H9	120.0	C22—C23—N5	116.8 (2)
C11—C10—C9	120.9 (3)	C18—C23—N5	117.7 (3)
C11—C10—H10	119.5	C17—N1—C14	107.3 (2)
C9—C10—H10	119.5	C17—N1—C7	111.5 (2)
C12—C11—C10	119.1 (3)	C14—N1—C7	111.4 (2)
C12—C11—H11	120.5	C16—N2—C15	110.5 (2)
C10—C11—H11	120.5	C16—N2—H2A	109.5
C11—C12—C13	120.6 (3)	C15—N2—H2A	109.5
C11—C12—H12	119.7	C16—N2—H2B	109.5
C13—C12—H12	119.7	C15—N2—H2B	109.5
C8—C13—C12	120.8 (3)	H2A—N2—H2B	108.1
C8—C13—H13	119.6	O3—N3—O2	120.7 (3)
C12—C13—H13	119.6	O3—N3—C19	119.5 (3)
N1—C14—C15	110.2 (2)	O2—N3—C19	119.1 (3)
N1—C14—H14A	109.6	O5—N4—O4	122.8 (3)
C15—C14—H14A	109.6	O5—N4—C21	118.5 (3)
N1—C14—H14B	109.6	O4—N4—C21	118.7 (2)
C15—C14—H14B	109.6	O6—N5—O7	124.4 (3)
H14A—C14—H14B	108.1	O6—N5—C23	117.7 (3)
N2—C15—C14	111.0 (2)	O7—N5—C23	117.8 (3)
N2—C15—H15A	109.4	H1WA—O1W—H1WA	0.0
C14—C15—H15A	109.4	H1WA—O1W—H1WB	111.4
N2—C15—H15B	109.4	H1WA—O1W—H1WB	111.4
C14—C15—H15B	109.4		

### Hydrogen-bond geometry (Å, º)

**Table:**

*D*—H···*A*	*D*—H	H···*A*	*D*···*A*	*D*—H···*A*
N2—H2*A*···O1*W*^i^	0.90	2.03	2.850 (4)	151
N2—H2*B*···O1^ii^	0.90	1.96	2.819 (3)	160
N2—H2*B*···O2^ii^	0.90	2.42	3.017 (4)	124
O1*W*—H1*WA*···O1	0.85	1.97	2.805 (3)	168
C16—H16*B*···O5^iii^	0.97	2.34	3.166 (4)	143

Symmetry codes: (i) −*x*+3/2, −*y*+1/2, −*z*+1; (ii) *x*, *y*, *z*−1; (iii) −*x*+2, −*y*+1, −*z*+1.
